# Supplementary material for: Deep Eutectic Solvents Based on Carboxylic Acids and Glycerol or Propylene Glycol as Green Media for Extraction of Bioactive Substances from Chamaenerion angustifolium (L.) Scop
Source: Molecules. 2023 Oct 8;28(19):6978. doi: 10.3390/molecules28196978 (PMC10574672; doi:10.3390/molecules28196978)
Supplement: Supplementary file 1 [file molecules-28-06978-s001.zip › molecules-2625263-supplementary.pdf]

Deep eutectic solvents based on carboxylic acids and glycerol or propylene glycol as green media for extraction of bioactive substances from *Chamanerion angustifolium* (L.) Scop.

Alena Koigerova [1], Alevtina Gosteva [2], Artemiy Samarov [3], and Nikita Tsvetov [1,2,] *

[1] Federal Research Centre "Kola Science Centre of the Russian Academy of Sciences", Laboratory of medical and biological technologies, Fersmana str. 14, Apatity, Russia; a.koygerova@ksc.ru
[2] I.V. Tananaev Institute of Chemistry and Technology of Rare Elements and Mineral Raw Materials - Subdivision of the Federal Research Centre «Kola Science Centre of the Russian Academy of Sciences», Akademgorodok 26a, Apatity, Russia; tsvet.nik@mail.ru; angosteva@list.ru
[3] Saint Petersburg State University, Institute of Chemistry, Department of Chemical Thermodynamics and Kinetics, Universitetskiy prosp. 26, St. Petersburg, Russia, samarov@yandex.ru
* Correspondence: tsvet.nik@mail.ru

Supplementary materials

S1. Experimental values of density and dynamic viscosity of suited DESs with 10 molar part of water at 30°C.

	Density	*Dyn. Viscosity*	*Variation Coefficient*	*Fw/Bw Deviation*
MAGL	1.16288	3.460	0.02	0.18
MAPG	1.10282	4.671	0.01	0.11
MalGL	1.20588	9.506	0.03	0.09
MalPG	1.13224	7.446	0.05	0.15
CAGL	1.23641	25.839	0.01	0.23
CAPG	1.13597	15.782	0.00	0.26

S2. Kinetical curves and linearization for TPC. 1-2 – MAGL, 3-4 – MAPG, 5-6 – MalGL, 7-8 –MalPG, 9-10 – CAGL, 11-12 – CAPG.

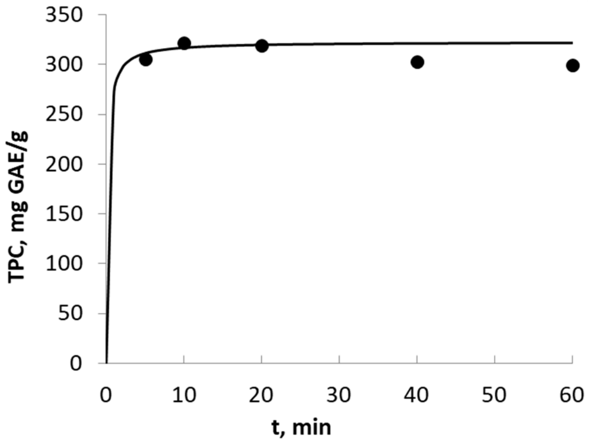

1)

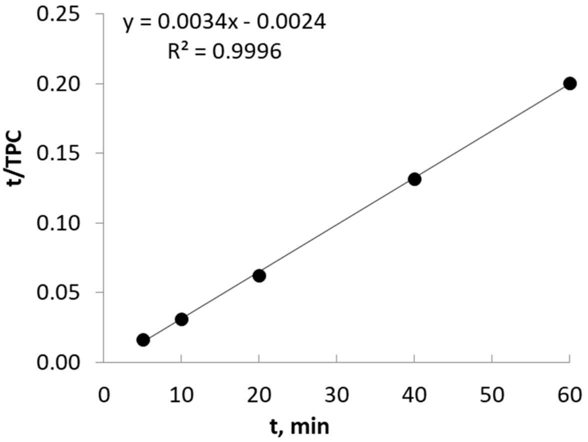

2)

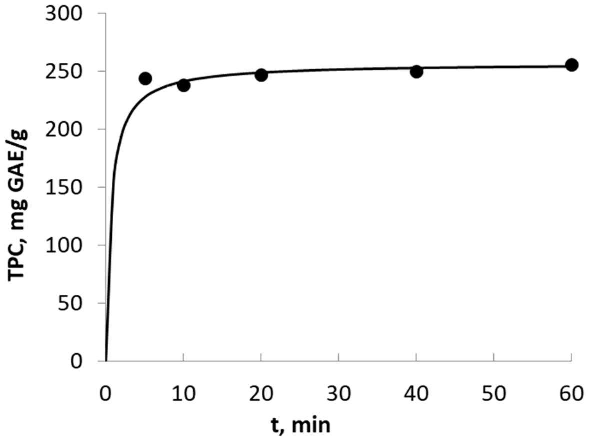

3)

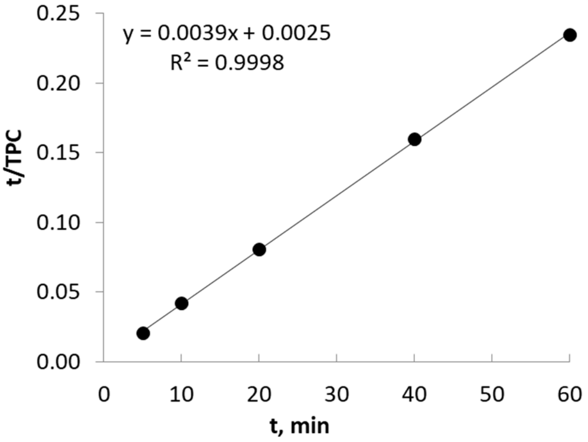

4)

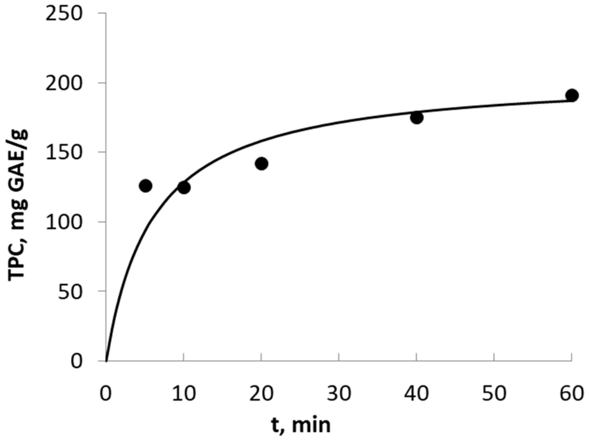

5)

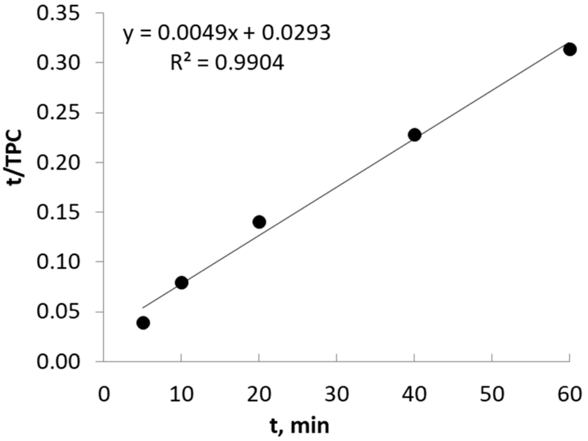

6)

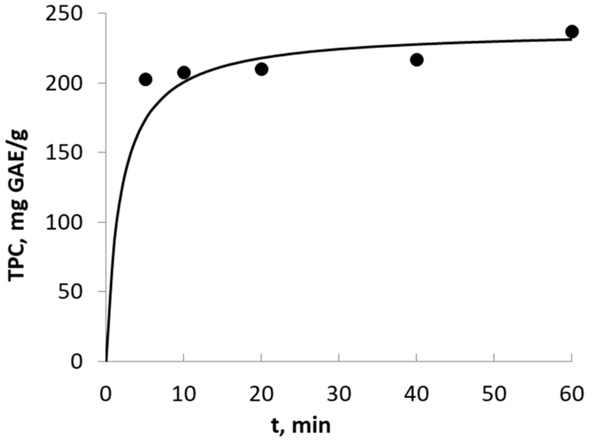
TPC, mg GAE/g
t, min

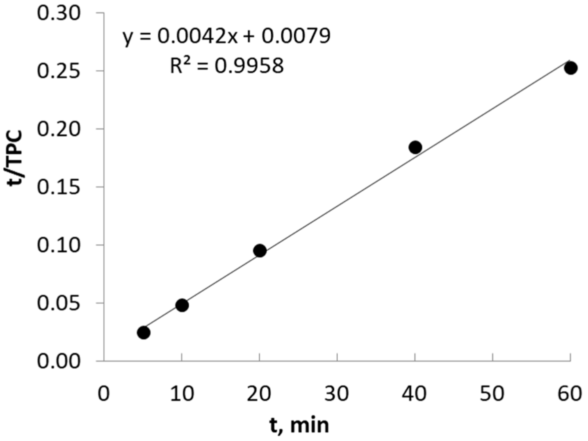
y = 0.0042x + 0.0079
R² = 0.9958
t/TPC
t, min

7)

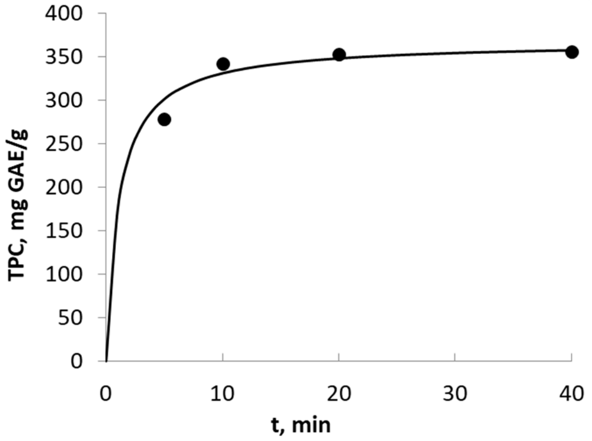
TPC, mg GAE/g
t, min

8)

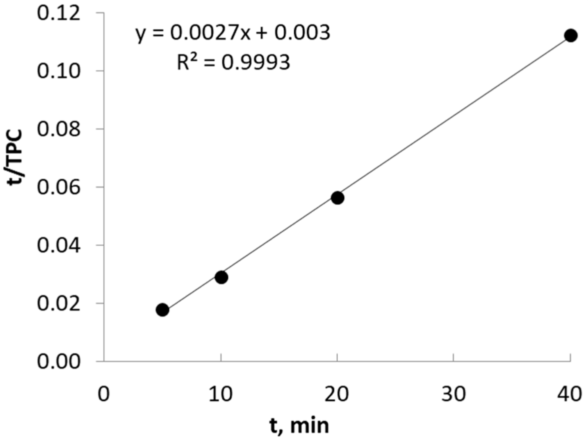
y = 0.0027x + 0.003
R² = 0.9993
t/TPC
t, min

9)

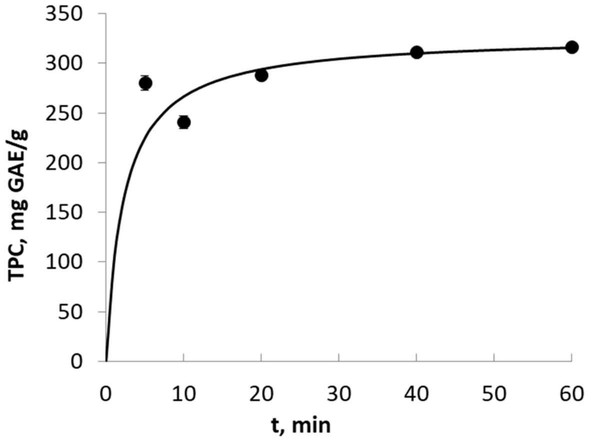
TPC, mg GAE/g
t, min

10)

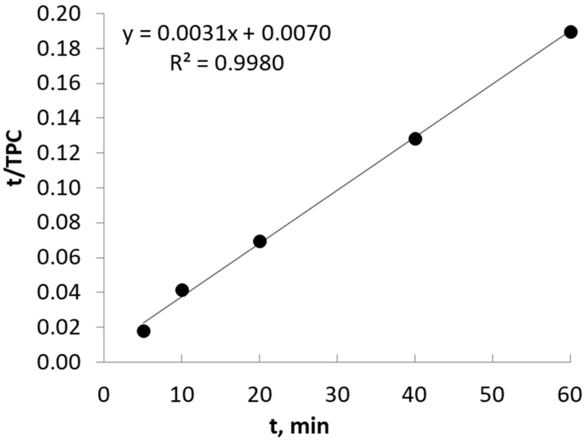
y = 0.0031x + 0.0070
R² = 0.9980
t/TPC
t, min

11)

12)

S3. Kinetical curves and linearization for TFC. 1-2 – MAGL, 3-4 – MAPG, 5-6 – MalGL, 7-8 –MalPG, 9-10 – CAGL, 11-12 – CAPG.

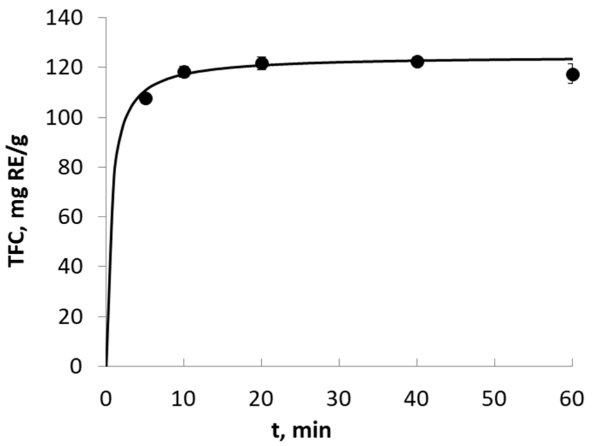

1)

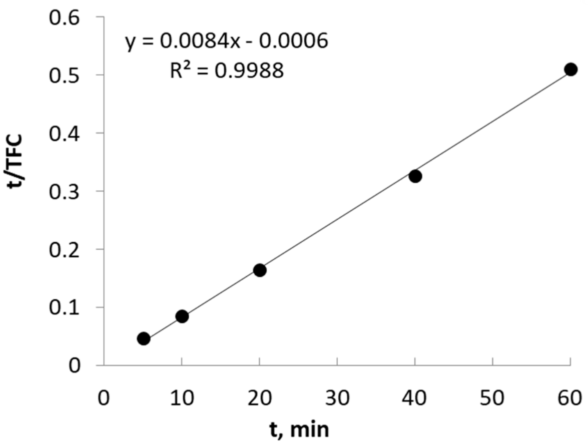

2)

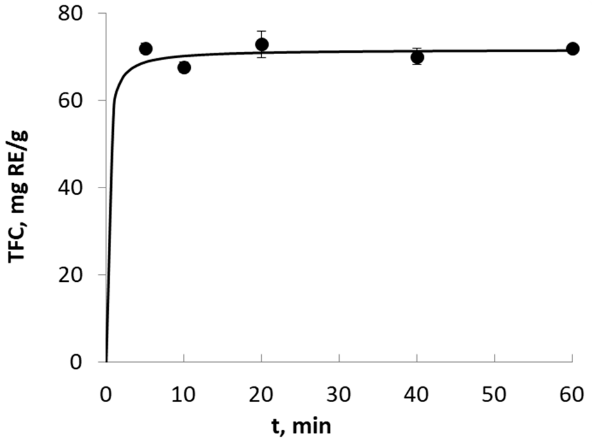

3)

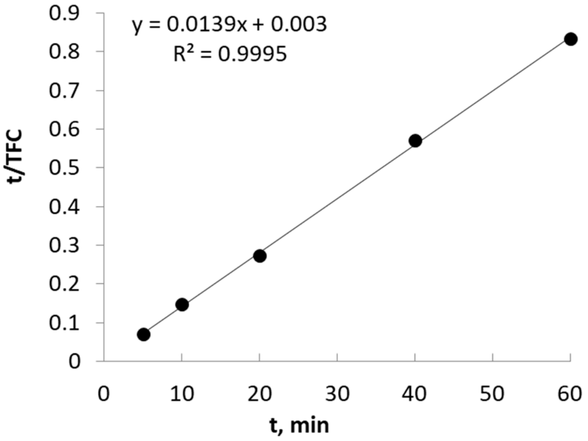

4)

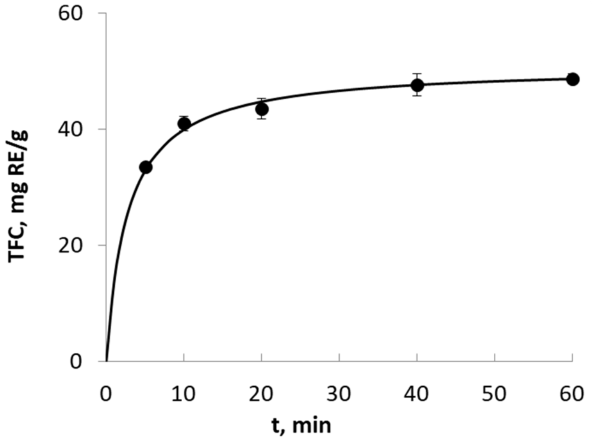

5)

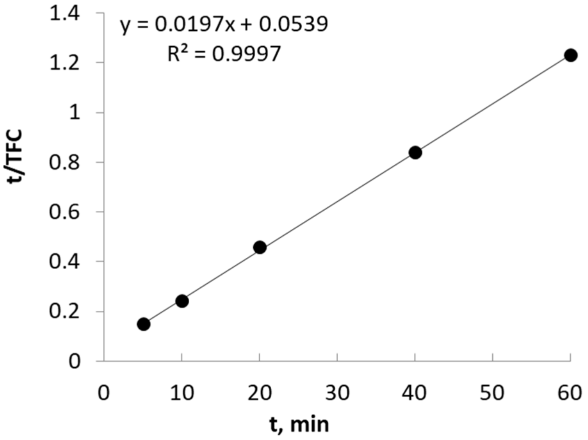

6)

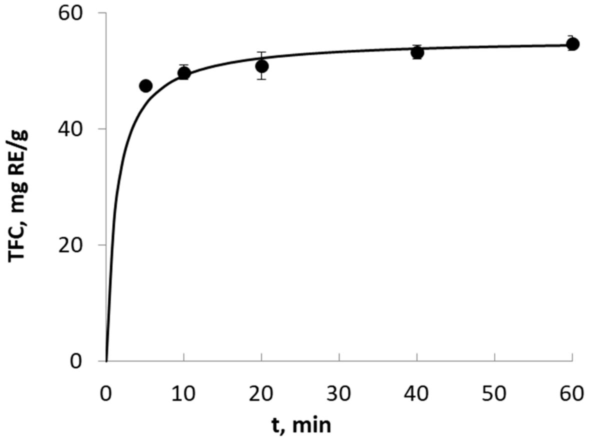
TFC, mg RE/g
t, min

7)

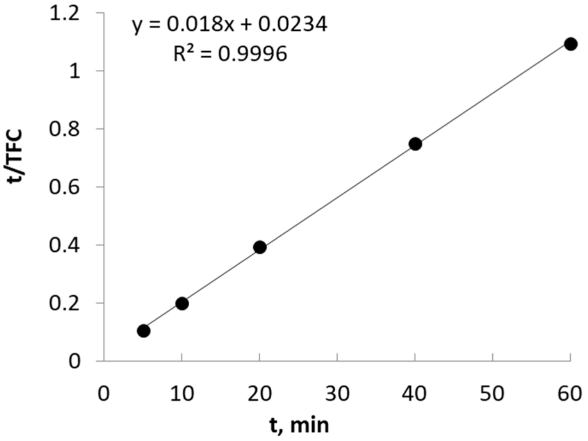
y = 0.018x + 0.0234
R² = 0.9996
t/TFC
t, min

8)

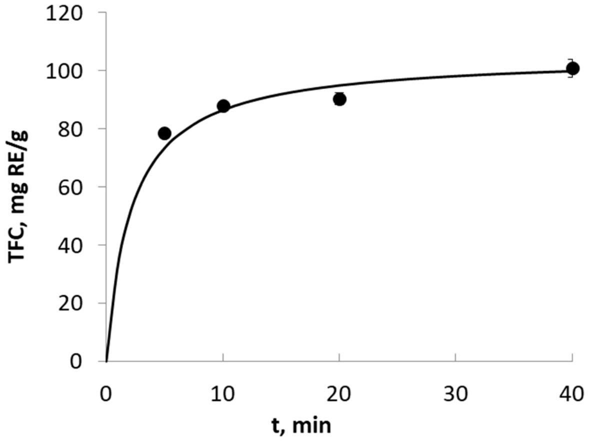
TFC, mg RE/g
t, min

9)

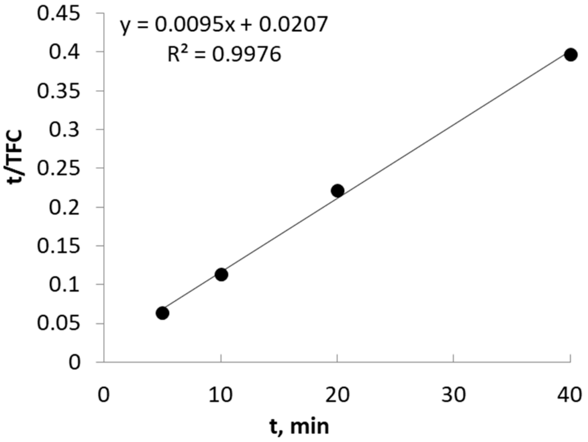
y = 0.0095x + 0.0207
R² = 0.9976
t/TFC
t, min

10)

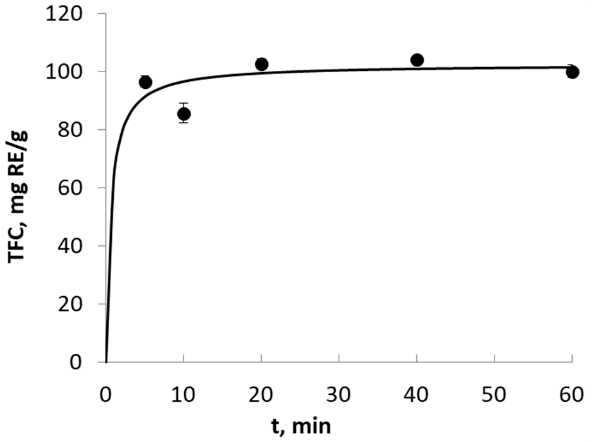
TFC, mg RE/g
t, min

11)

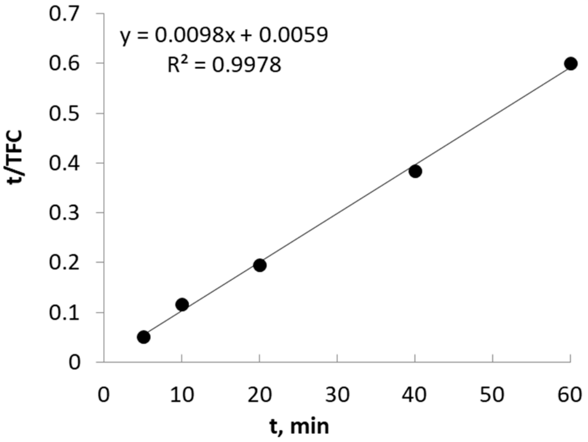
y = 0.0098x + 0.0059
R² = 0.9978
t/TFC
t, min

12)

S4. Calculated equilibrium yiels (Y^{eq}) and rate constants (k) for TPC and TFC.

DES	TPC, mg GAE/g		TFC, mg RE/g	
	Y^{eq}	$k\times10^3$	Y^{eq}	$k\times10^3$
MAGL	322.7	17.0	124.7	12.7
MAPG	257.0	6.0	71.7	65.6
MalGL	205.9	0.8	50.9	7.2
MalPG	238.4	2.2	55.6	13.8
CAGL	367.3	2.5	105.2	4.4
CAPG	327.7	1.3	102.5	16.0

S5. Kinetical curves and linearization for TAC. 1-2 – MAGL, 3-4 – MAPG, 5-6 – MalGL, 7-8 –MalPG, 9-10 – CAGL, 11-12 – CAPG.

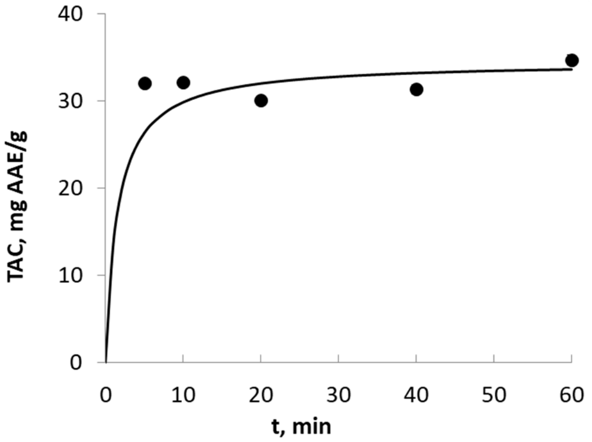

1)

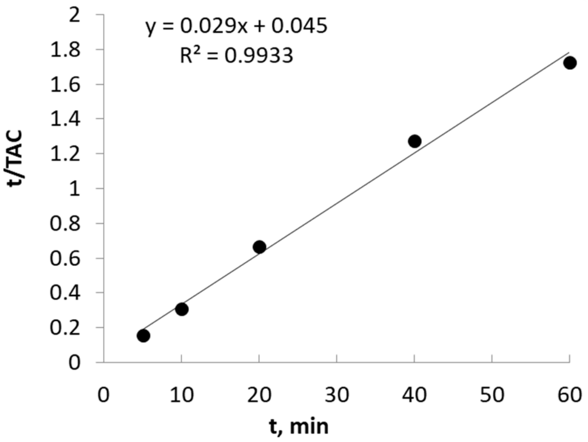

2)

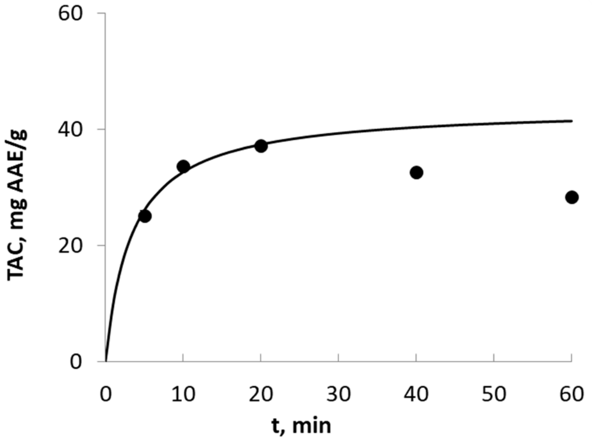

3)

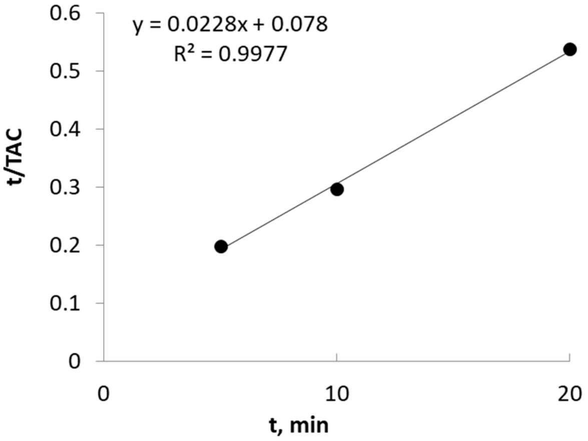

4)

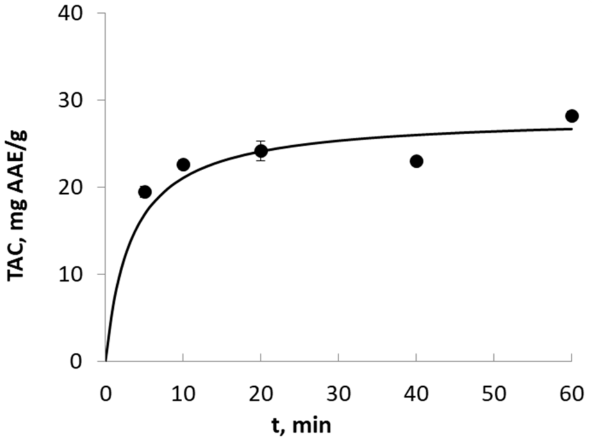

5)

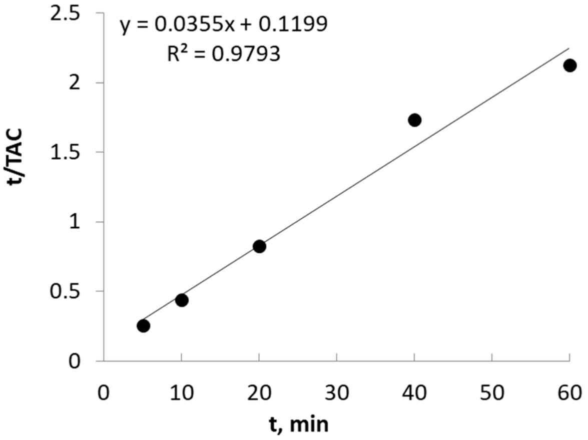

6)

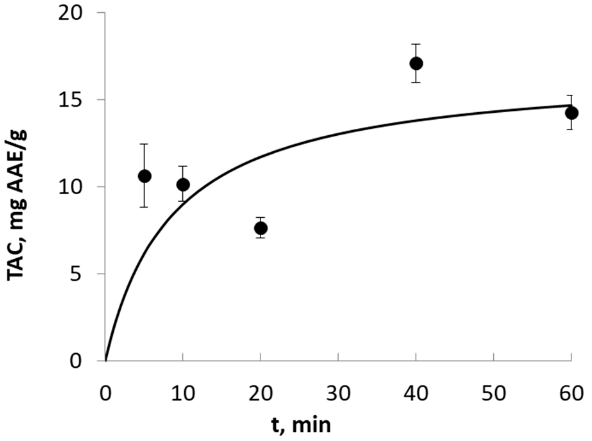
TAC, mg AAE/g
t, min

7)

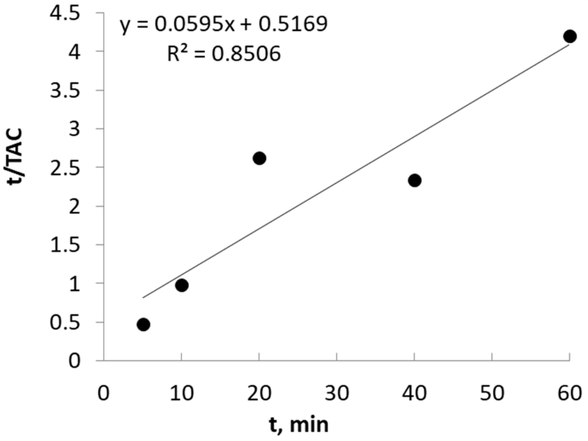
y = 0.0595x + 0.5169
R² = 0.8506
t/TAC
t, min

8)

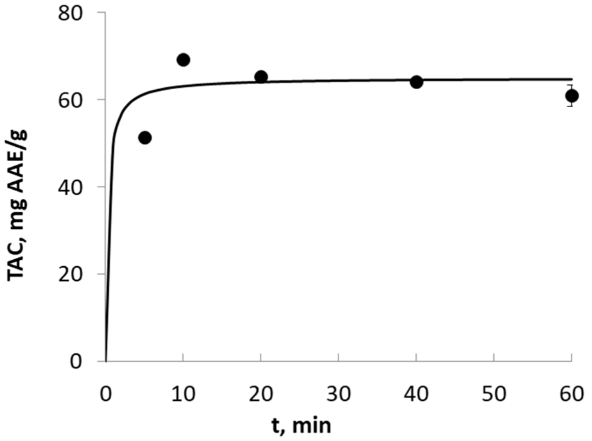
TAC, mg AAE/g
t, min

9)

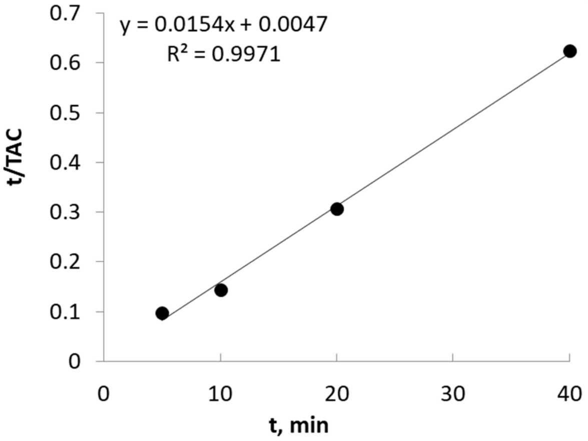
y = 0.0154x + 0.0047
R² = 0.9971
t/TAC
t, min

10)

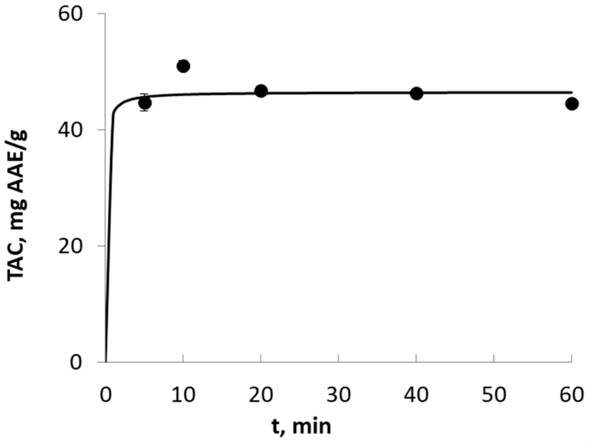
TAC, mg AAE/g
t, min

11)

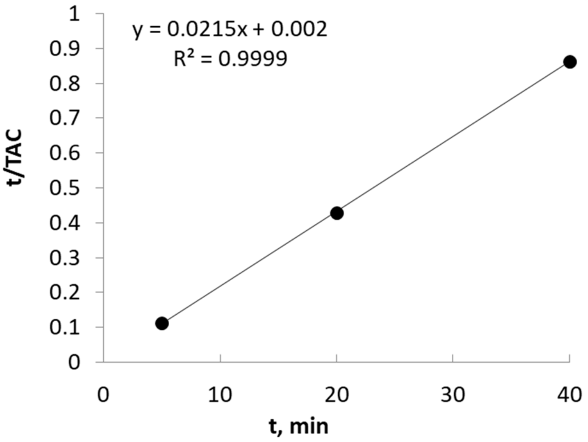
y = 0.0215x + 0.002
R² = 0.9999
t/TAC
t, min

12)

S6. Kinetical curves and linearization for DPPH. 1-2 – MAGL, 3-4 – MAPG, 5-6 – MalGL, 7-8 –MalPG, 9-10 – CAGL, 11-12 – CAPG.

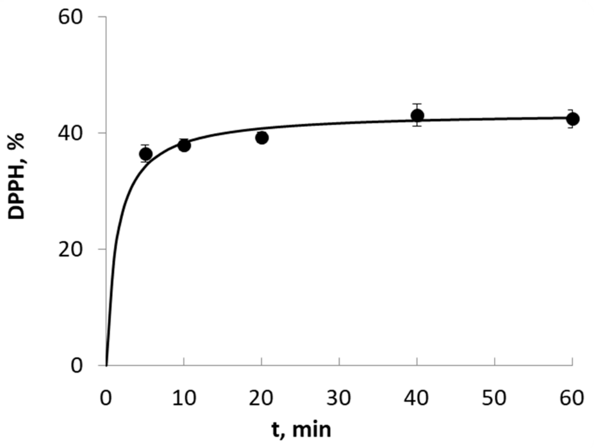

1)

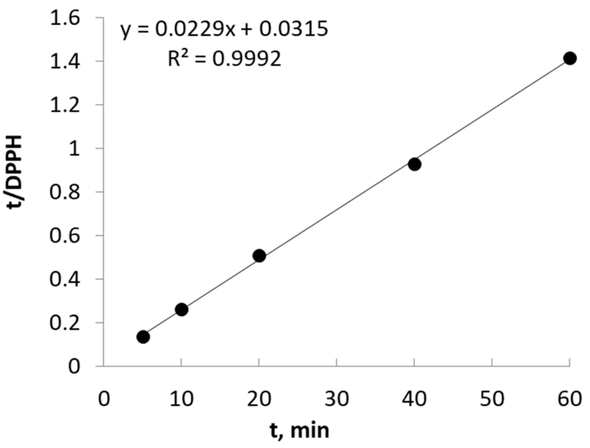

2)

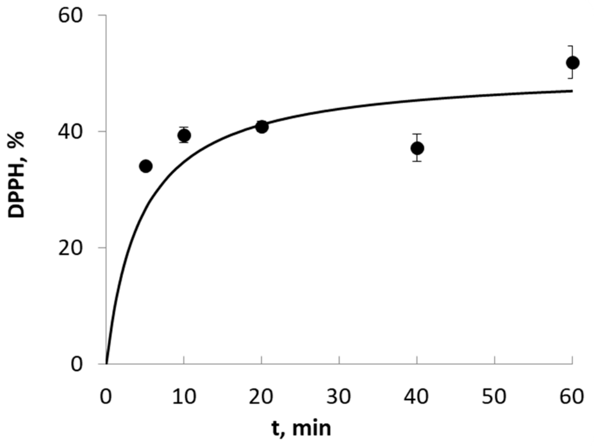

3)

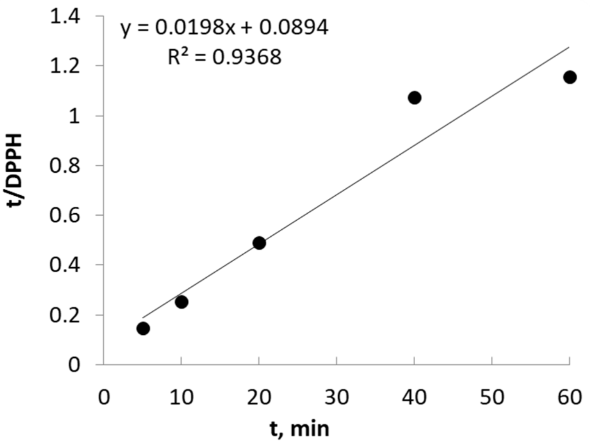

4)

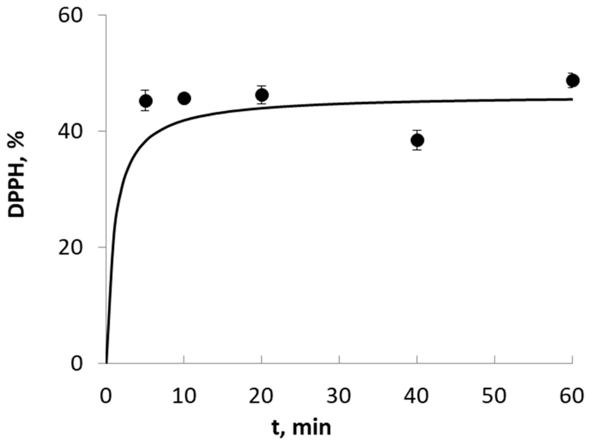

5)

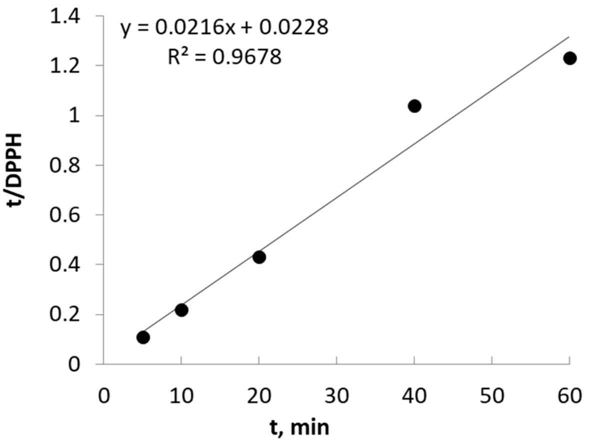

6)

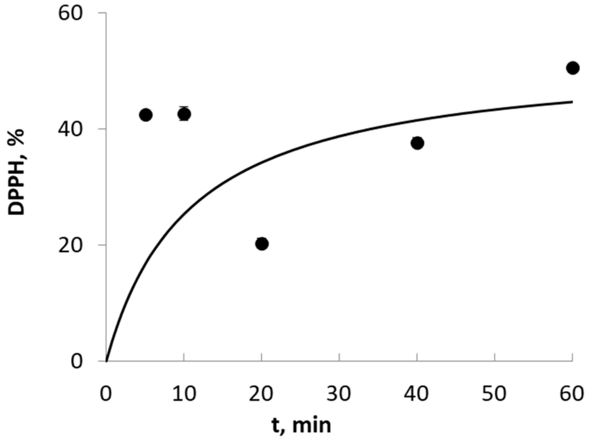
DPPH, %
t, min

7)

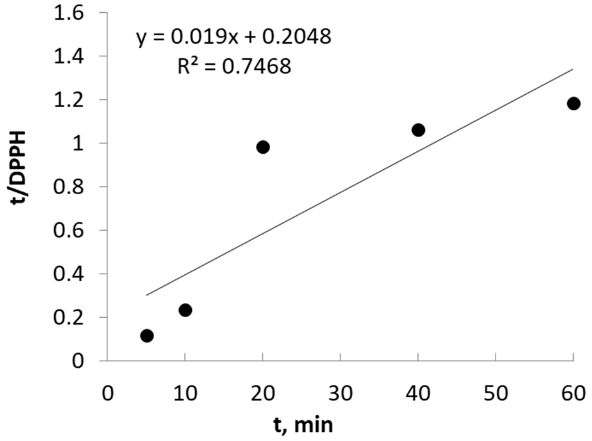
y = 0.019x + 0.2048
R² = 0.7468
t/DPPH
t, min

8)

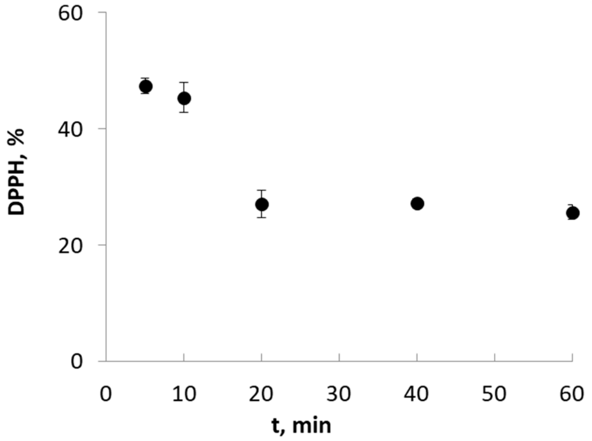
DPPH, %
t, min

9)

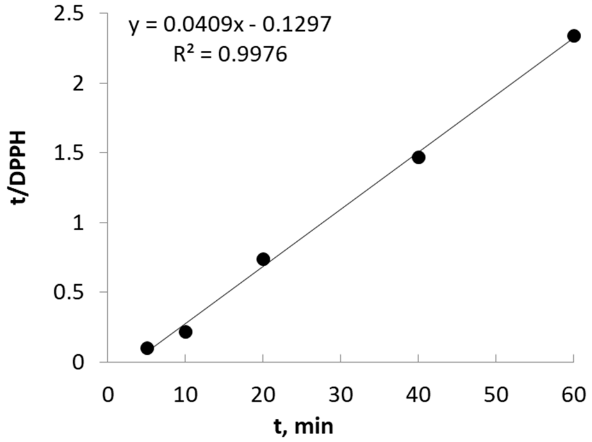
y = 0.0409x - 0.1297
R² = 0.9976
t/DPPH
t, min

10)

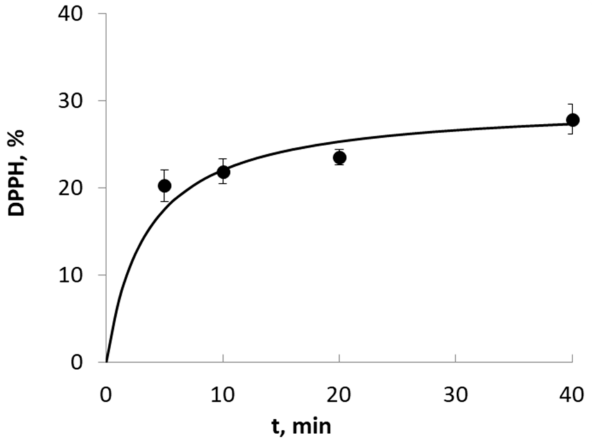
DPPH, %
t, min

11)

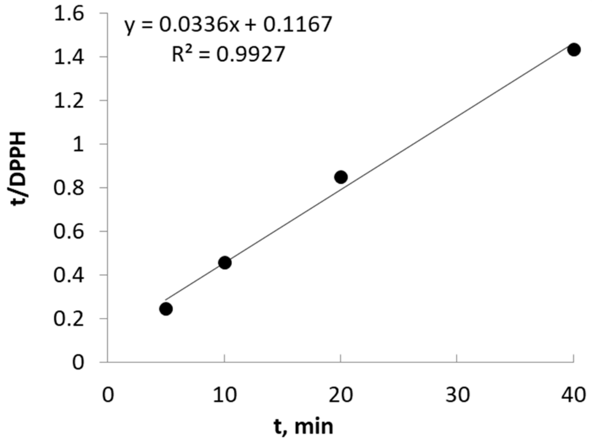
y = 0.0336x + 0.1167
R² = 0.9927
t/DPPH
t, min

12)

S7. Calculated equilibrium yiels (Y^{eq}) and rate constants (k) for TAC and DPPH.

**DES**	**TAC, mg AAE/g**		**DPPH, %**	
	$\boldsymbol{Y^{eq}}$	$\boldsymbol{k\times10^3}$	$\boldsymbol{Y^{eq}}$	$\boldsymbol{k\times10^3}$
MAGL	34.5	18.7	43.6	16.7
MAPG	43.8	6.7	50.5	4.4
MalGL	28.2	10.5	46.3	20.4
MalPG	16.8	6.9	52.7	1.8
CAGL	65.0	50.5	25.0	-13.6
CAPG	46.5	233.3	29.7	9.7

S8. Kinetical curves and linearization for myricetin. 1-2 – MAGL, 3-4 – MAPG, 5-6 – MalGL, 7-8 –MalPG, 9-10 – CAGL, 11-12 – CAPG.

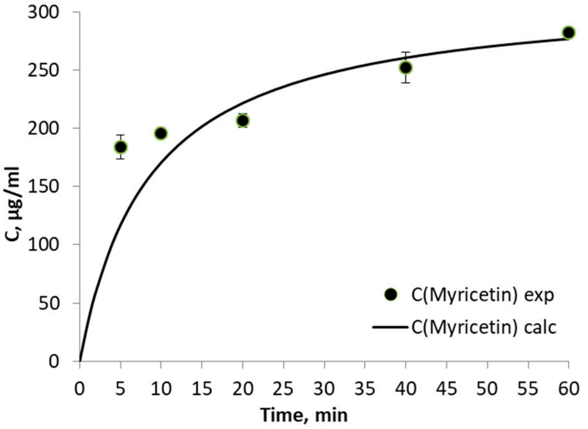

1)

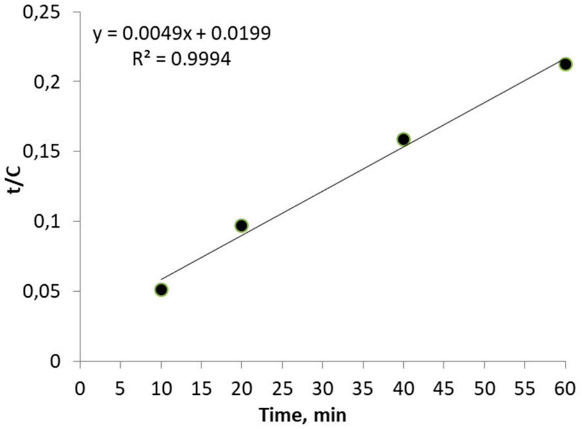

2)

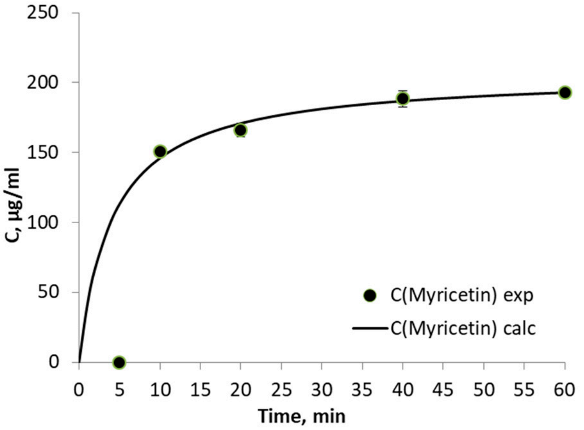

3)

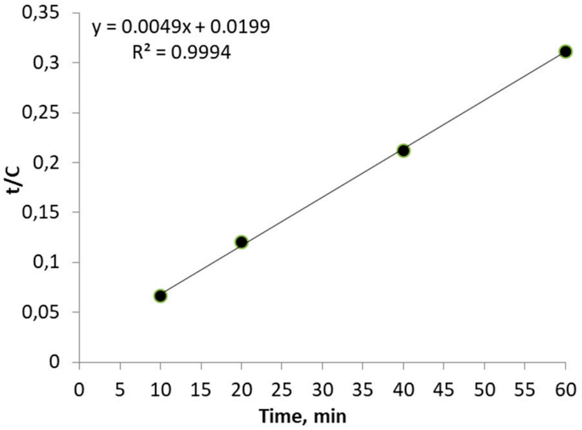

4)

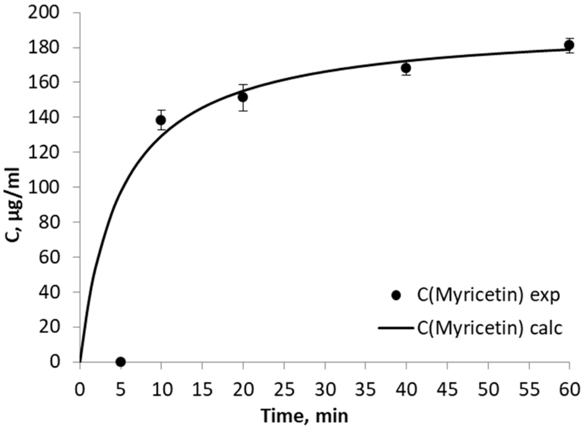

5)

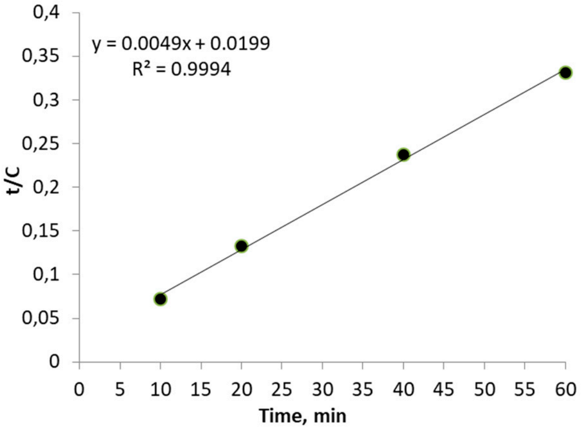

6)

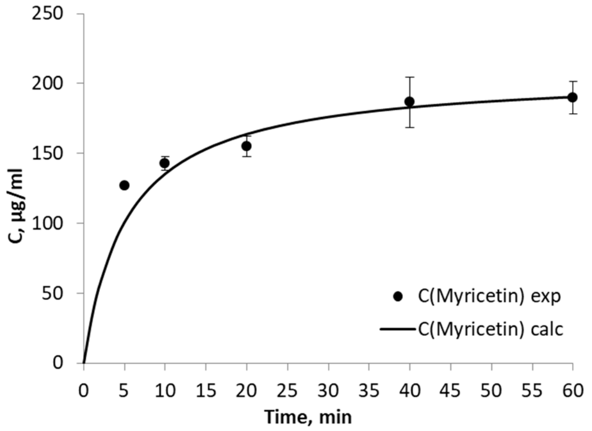
C, µg/ml
Time, min
C(Myricetin) exp
C(Myricetin) calc

7)

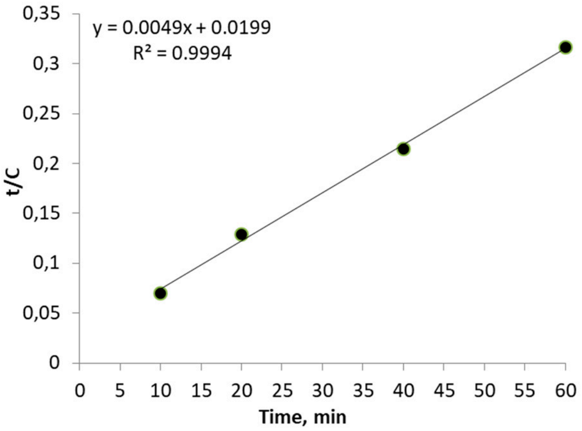
y = 0.0049x + 0.0199
R² = 0.9994
t/C
Time, min

8)

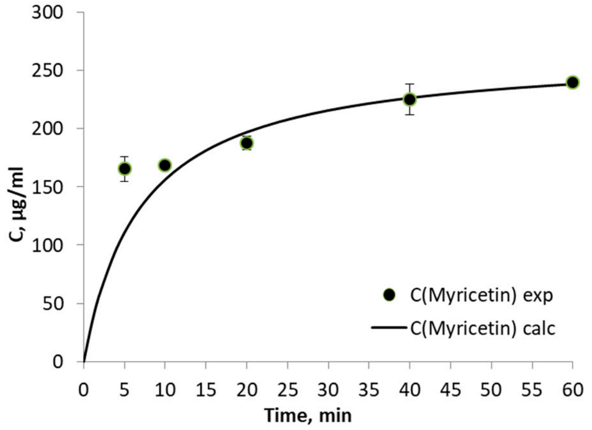
C, µg/ml
Time, min
C(Myricetin) exp
C(Myricetin) calc

9)

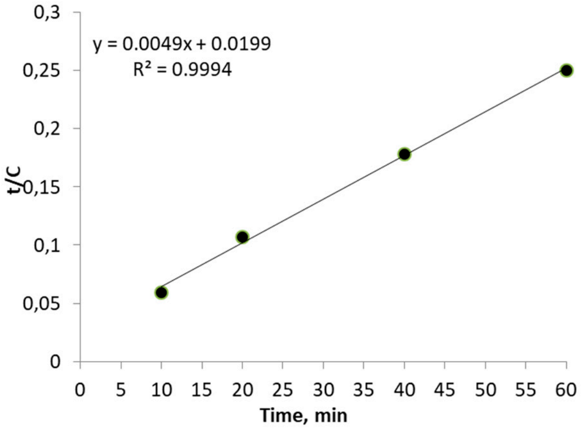
y = 0.0049x + 0.0199
R² = 0.9994
t/C
Time, min

10)

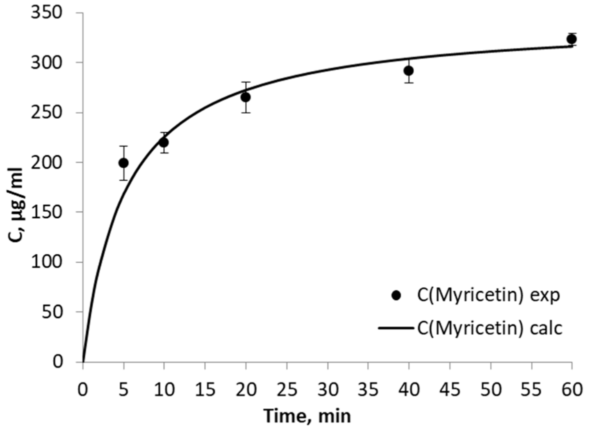
C, µg/ml
Time, min
C(Myricetin) exp
C(Myricetin) calc

11)

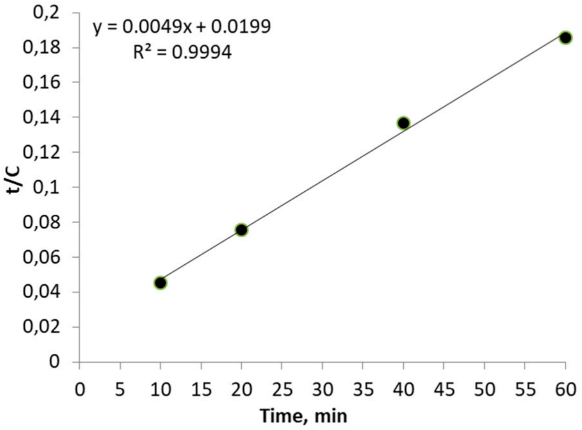
y = 0.0049x + 0.0199
R² = 0.9994
t/C
Time, min

12)

S9. Kinetical curves and linearization for quercetin. 1-2 – MAGL, 3-4 – MAPG, 5-6 – MalGL, 7-8 –MalPG, 9-10 – CAGL, 11-12 – CAPG.

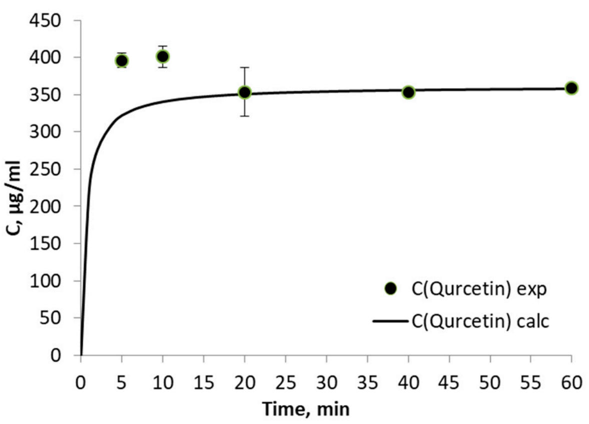

1)

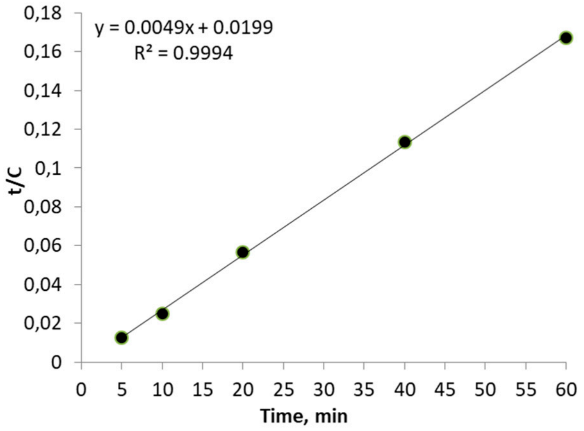

2)

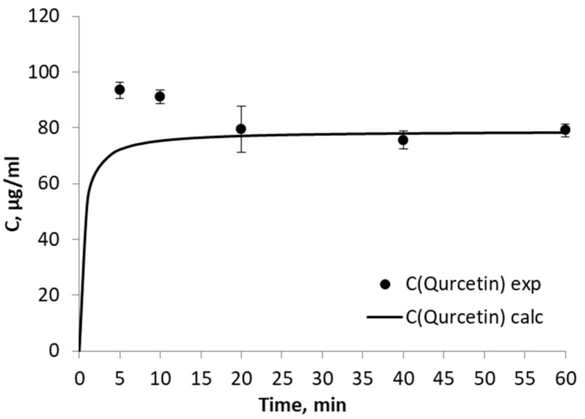

3)

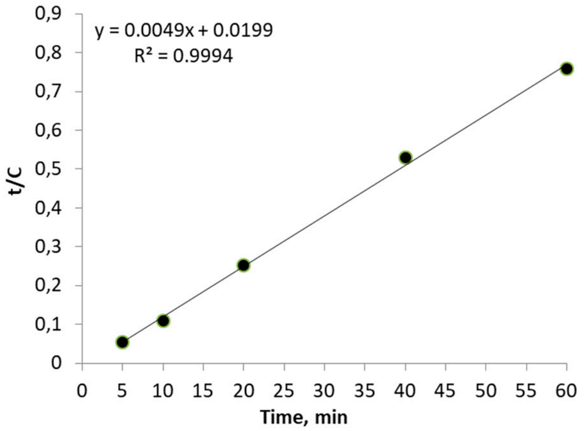

4)

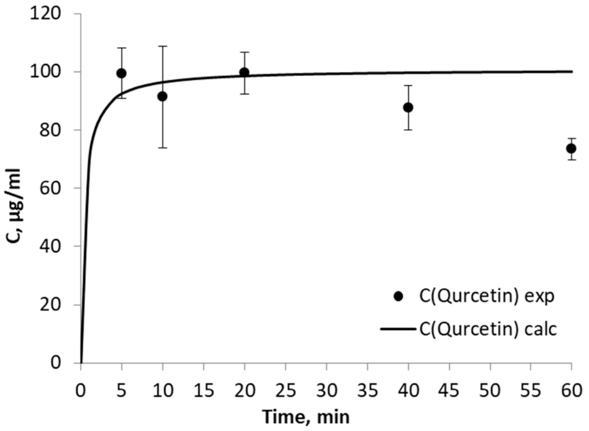

5)

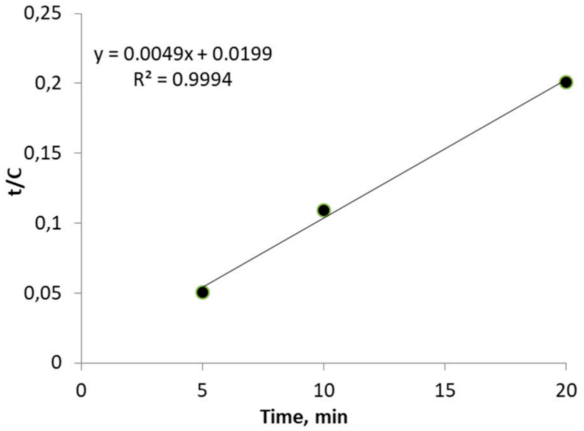

6)

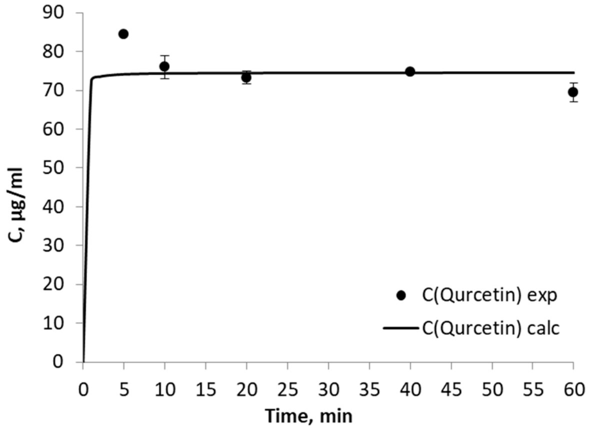
C, µg/ml
Time, min
C(Qurcetin) exp
C(Qurcetin) calc

7)

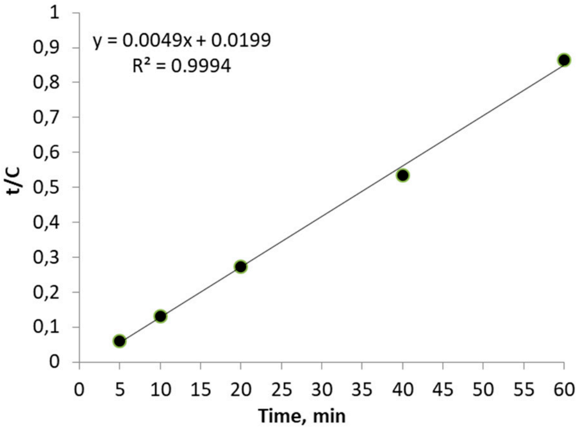
y = 0.0049x + 0.0199
R² = 0.9994
t/C
Time, min

8)

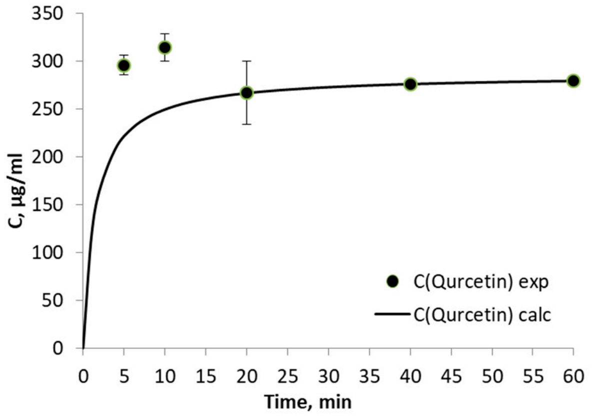
C, µg/ml
Time, min
C(Qurcetin) exp
C(Qurcetin) calc

9)

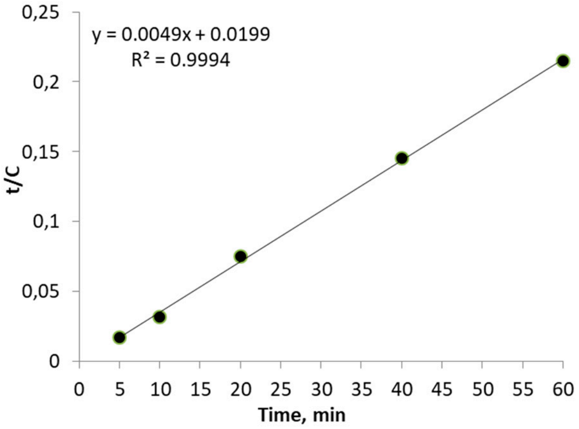
y = 0.0049x + 0.0199
R² = 0.9994
t/C
Time, min

10)

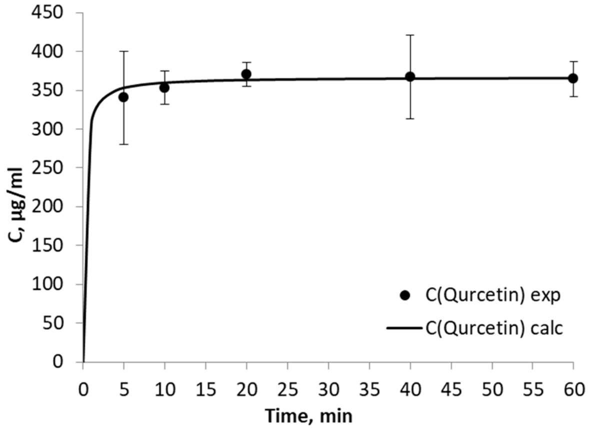
C, µg/ml
Time, min
C(Qurcetin) exp
C(Qurcetin) calc

11)

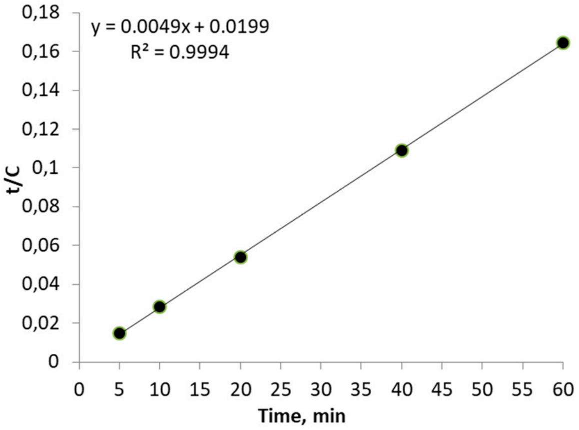
y = 0.0049x + 0.0199
R² = 0.9994
t/C
Time, min

12)

S10. Kinetical curves and linearization for kaempherol. 1-2 – MAGL, 3-4 – MAPG, 5-6 – MalGL, 7-8 –MalPG, 9-10 – CAGL, 11-12 – CAPG.

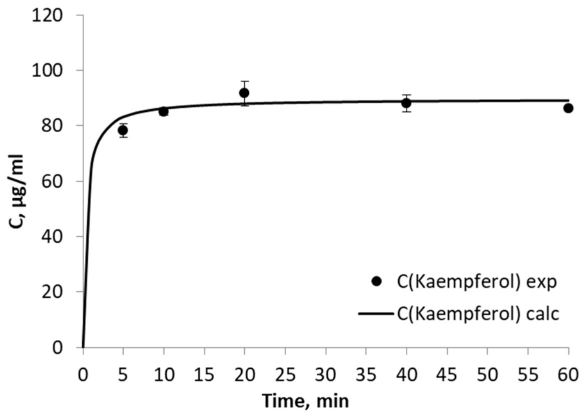

1)

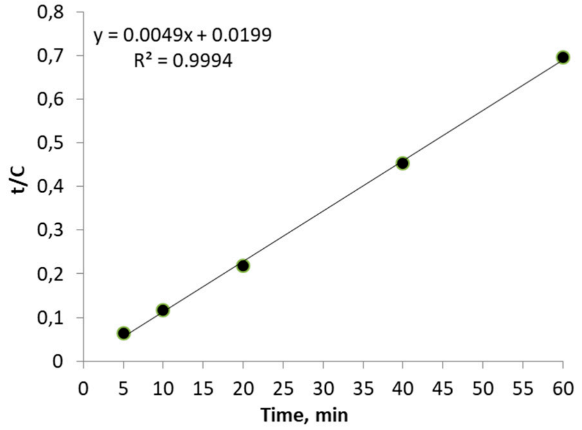

2)

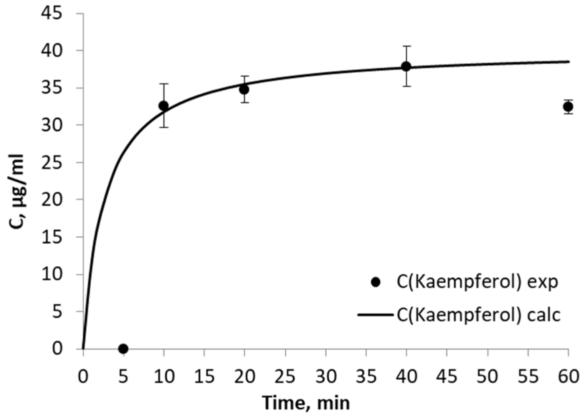

3)

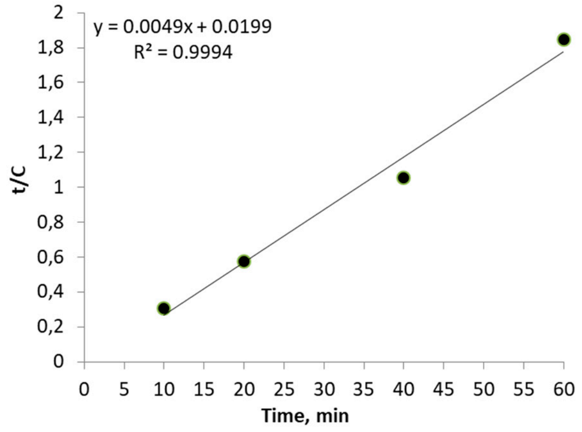

4)

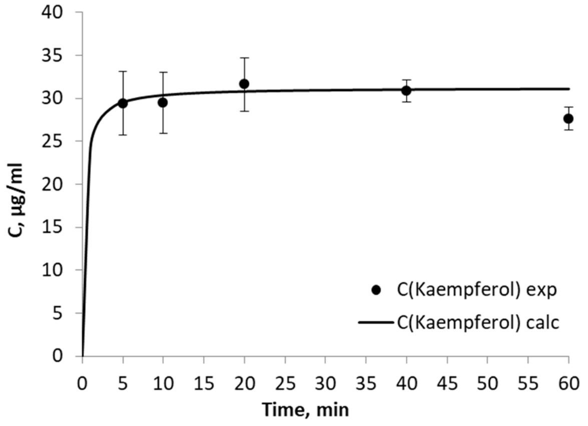

5)

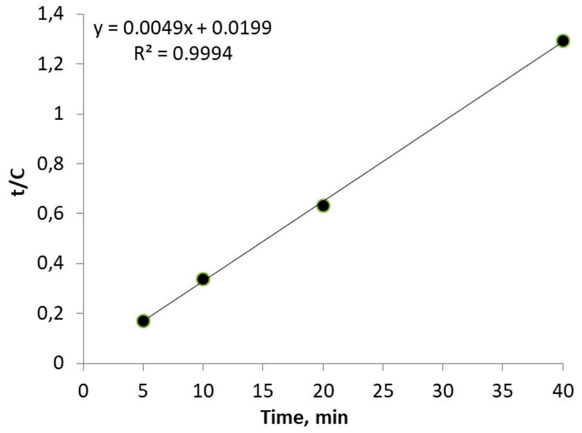

6)

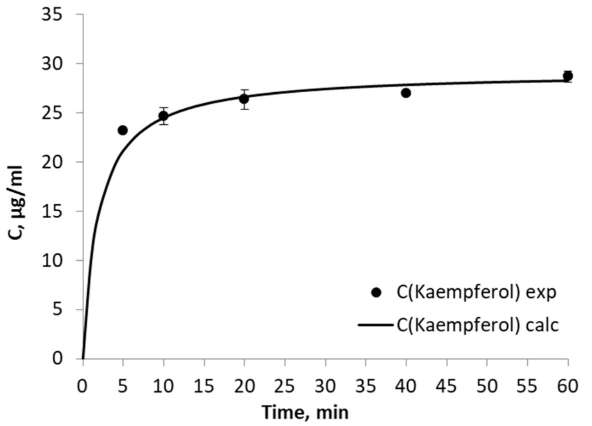
C, μg/ml
Time, min
C(Kaempferol) exp
C(Kaempferol) calc

7)

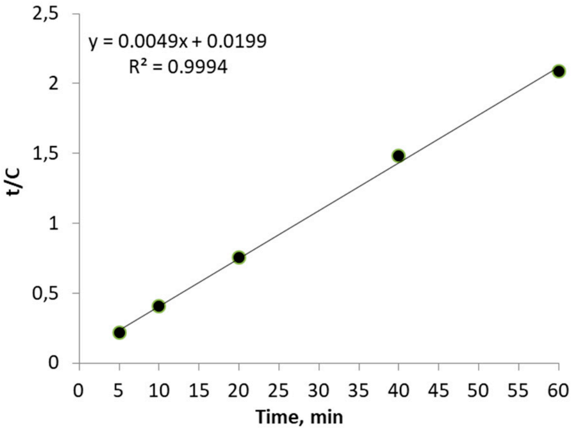
y = 0.0049x + 0.0199
R² = 0.9994
t/C
Time, min

8)

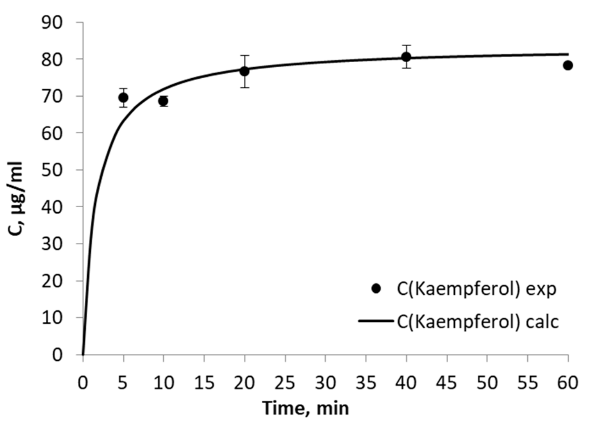
C, μg/ml
Time, min
C(Kaempferol) exp
C(Kaempferol) calc

9)

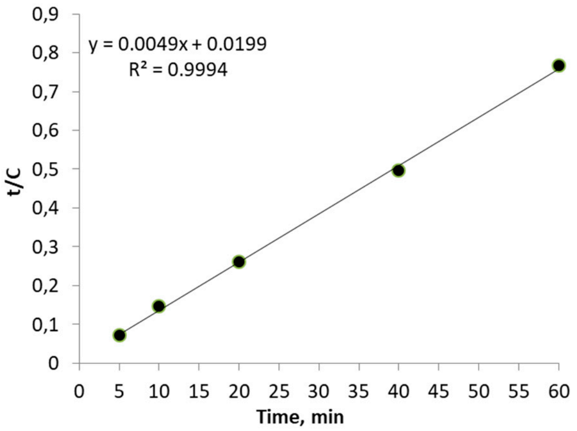
y = 0.0049x + 0.0199
R² = 0.9994
t/C
Time, min

10)

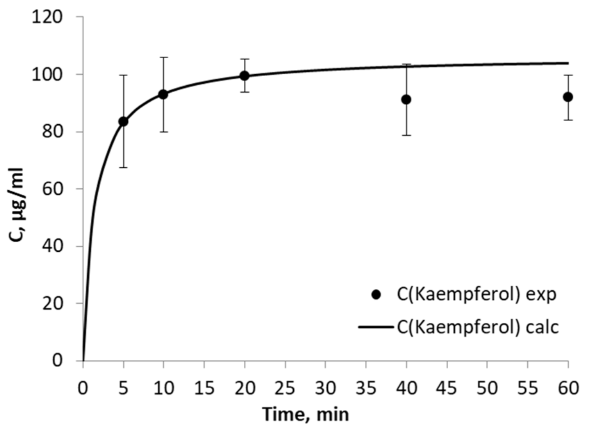
C, μg/ml
Time, min
C(Kaempferol) exp
C(Kaempferol) calc

11)

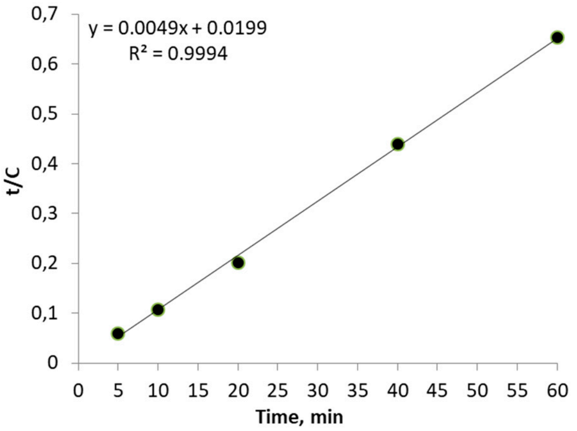
y = 0.0049x + 0.0199
R² = 0.9994
t/C
Time, min

12)

S11. Calculated equilibrium yiels (Y^{eq}) and rate constants (k) for describing of the experimental data on the kinetics of myricetin, quercetin , and kaempherol glycosides extraction.

**DES**	**c(myricetin)**		**c(quercetin)**		**c(kaempferol)**	
	Y^{eq}	$k\times10^3$	Y^{eq}	$k\times10^3$	Y^{eq}	$k\times10^3$
MAGL	316.0	0.4	361.7	4.4	89.7	28.3
MAPG	206.1	1.2	78.9	27.0	40.2	9.4
MalGL	193.6	1.0	100.8	21.9	31.3	111.1
MalPG	207.2	0.9	74.6	464.9	29.2	18.0
CAGL	266.0	0.5	285.9	2.4	83.5	7.5
CAPG	343.7	0.6	366.9	13.9	106.4	6.7
